# Comparison of Image-Guided Intensity-Modulated Radiotherapy and Low-dose Rate Brachytherapy with or without External Beam Radiotherapy in Patients with Localized Prostate Cancer

**DOI:** 10.1038/s41598-018-28730-1

**Published:** 2018-07-12

**Authors:** Takuji Tsubokura, Hideya Yamazaki, Koji Masui, Naomi Sasaki, Daisuke Shimizu, Gen Suzuki, Satoaki Nakamura, Kei Yamada, Koji Okihara, Takumi Shiraishi, Ken Yoshida, Tatsuyuki Nishikawa, Haruumi Okabe

**Affiliations:** 1 Department of Radiology, Fukuchiyama City Hospital, 231 Atsunakamachi, Fukuchiyama, Kyoto Prefecture 620-8505 Japan; 2 Department of Radiology, Graduate School of Medical Science, Kyoto Prefectural University of Medicine, 465 Kajiicho Kawaramachi Hirokoji, Kamigyo-ku, Kyoto, 602-8566 Japan; 3 Urology, Graduate School of Medical Science, Kyoto Prefectural University of Medicine, 465 Kajiicho Kawaramachi Hirokoji, Kamigyo-ku, Kyoto, 602-8566 Japan; 4 Department of Radiology, Osaka Medical College, 2-7 Daigaku-machi, Takatsuki-City, Osaka 569-8686 Japan; 5 Department of Radiology, Ujitakeda Hospital, Uji-city, Kyoto Japan

## Abstract

To compare the outcome of low-dose rate brachytherapy (LDR-BT) and image-guided intensity-modulated radiotherapy (IG-IMRT) for localized prostate cancer, we examined 488 LDR-BT and 269 IG-IMRT patients. IG-IMRT treated older and advanced disease with more hormonal therapy than LDR-BT, which excluded T3b–T4 tumor and initial PSA > 50 ng/ml. The actuarial five-year biochemical failure-free survival rate was 88.7% and 96.7% (*p* = 0.0003) in IG-IMRT and LDR-BT, respectively; it was 88.2% (85.1% for IG-IMRT and 94.9% for LDR-BT, *p* = 0.0578) for the high-risk group, 95.2% (91.6% and 97.0%, *p* = 0.3361) for the intermediate IG-IMRT and 96.8% (95.7% and 97%, *p* = 0.8625) for the low-risk group. Inverse probability of treatment weighting (IPTW) involving propensity scores was used to reduce background selection bias. IPTW showed a statistically significant difference between LDR-BT and IG-IMRT in high risk (p = 0.0009) and high risk excluding T3-4/initial PSA > 50 ng/ml group (p = 0.0073). IG-IMRT showed more gastrointestinal toxicity (p = 0.0023) and less genitourinary toxicity (p < 0.0001) than LDR-BT. LDR-BT and IG-IMRT showed equivocal outcome in low- and intermediate-risk groups. For selected high-risk patients, LDR-BT showed more potential to improve PSA control rate than IG-IMRT.

## Introduction

Although prostate cancer is one of the most prevalent cancers in men, only 10–15% of patients die from prostate cancer^1^. Most men with prostate cancer die from other causes^2^. Therefore, many tactics for treatment of prostate cancer exist depending on their disease staging, age, performance status, and patients’ preferences’^3^. Surgery, radiotherapy, external beam radiotherapy (EBRT), brachytherapy, hormonal therapy, or a combination of those therapies and even watchful waiting is also applicable to the elderly or fragile patient^3^.

Recently, image-guided intensity-modulated radiotherapy (IG-IMRT) has been widely used for prostate cancer. Because IG-IMRT is able to further reduces the adverse events more than three-dimensional conformal radiotherapy (3D-CRT) and even IMRT^4^, we have installed IG-IMRT using helical tomotherapy with or without hormonal therapy, which permits precise dose delivery using megavoltage-computed tomography (MVCT)^5,6^. Advanced EBRT has become one of the standard treatments for all stages of localized prostate cancer based on confirmed evidence^3^.

In addition, although an invasive procedure is required, we have also employed a low-dose rate brachytherapy (LDR-BT) because brachytherapy has a clear advantage; i.e., excellent dose distribution using shallow-dose gradient around radioactive source and precise dose delivery to the tumor by direct insertion, which enables organ motion nearly negligible^7^. In our institution, the LDR-BT was initially applied for the low-risk group. Thereafter, LDR-BT’s application was expanded to intermediate- to high-risk patients to enhance the merit of delivering higher irradiation dose to the tumor, which has the potential to improve tumor control^8^.

To date, several trials including randomized controlled trials have demonstrated the benefits of biochemical control benefits by dose-escalation^3,4^. However, little evidence exists directly comparing the effectiveness of modern IG-IMRT and LDR-BT. In the absence of matured randomized controlled trial, pairing patients with known and matching prognostic factors can be an alternative method for exploring differences in patients’ outcome between treatments. Therefore, we introduced inverse probability of treatment weighting (IPTW) involving propensity scores to reduce background selection bias. The aim of this study is to compare the outcome of IG-IMRT and LDR-BT and examine its rationale based on current clinical outcomes.

## Results

### Patients characteristics

The median follow-up for the entire cohort was 68.0 (ranging from 12–121) months. A comparison of the two schedule backgrounds is shown in Table 1. IG-IMRT treated older and advanced disease with more hormonal therapy and longer follow-up periods than LDR-BT. LDR-BT actually excluded T3b–T4 tumors from indication, and no patients had an initial PSA (iPSA) of 50 ng/ml or more (Table 1). In detailed subgroup analysis, there is no background difference between LDR-BT and IG-IMRT in low risk group (Supplemental Table 1), however, there remained background deviations in intermediate-, high-, and high risk group excluding T3b-4 or iPSA 50 ng/ml or more.

**Table 1:** Characteristics and treatment factors of patients.

Variables	Strata	IG-IMRT			LDR-BT		*p*-value
		n = 269			n = 488		
		No. or Median (range)	(%)		No. or Median (range)	(%)	
Age		71.5 (51–86)			71 (52–86)		**0.0147**
T category	1	86	(32%)		234	(48%)	**<0.0001**
	2	118	(44%)		242	(49%)	
	3	64	(24%)		12	(2%)	
	4	1	(0.4%)		0	(0%)	
iPSA	ng/ml	9.7 (4–265)			7.0 (1.4–46)		**<0.0001**
Gleason score	−6	86	(32%)		279	(57%)	**<0.0001**
	7	76	(28%)		185	(38%)	
	8−	107	(40%)		24	(5%)	
D’Amicos’ risk classification	Low	47	(17%)		193	(39%)	**<0.0001**
	Intermediate	72	(27%)		222	(45%)	
	High	150	(56%)		73	(15%)	
Prescribed dose	74.8 Gy	102	(38%)	LDR-BT 110 Gy plus EBRT 40 Gy	68	(14%)	NA
	72.6 Gy	23	(9%)	LDR-BT 145 Gy	420	(86%)	
	74 Gy	119	(44%)				
	72 Gy	25	(9%)				
Hormonal therapy	Yes	176	(65%)		156	(32%)	**<0.0001**
	No	93	(35%)		332	(68%)	
Follow-up	Months	74.3 (23.2–96)			60.5 (12–121.6)		**<0.0001**

*Bold values indicate statistically significance, NA; not available.

LDR-BT; low-dose-rate brachytherapy, EBRT: external beam radiothrapy

IG-IMRT; image guided intensity modulated radiotherapy.

### Biochemical control and survival outcome

**Figure 1:**
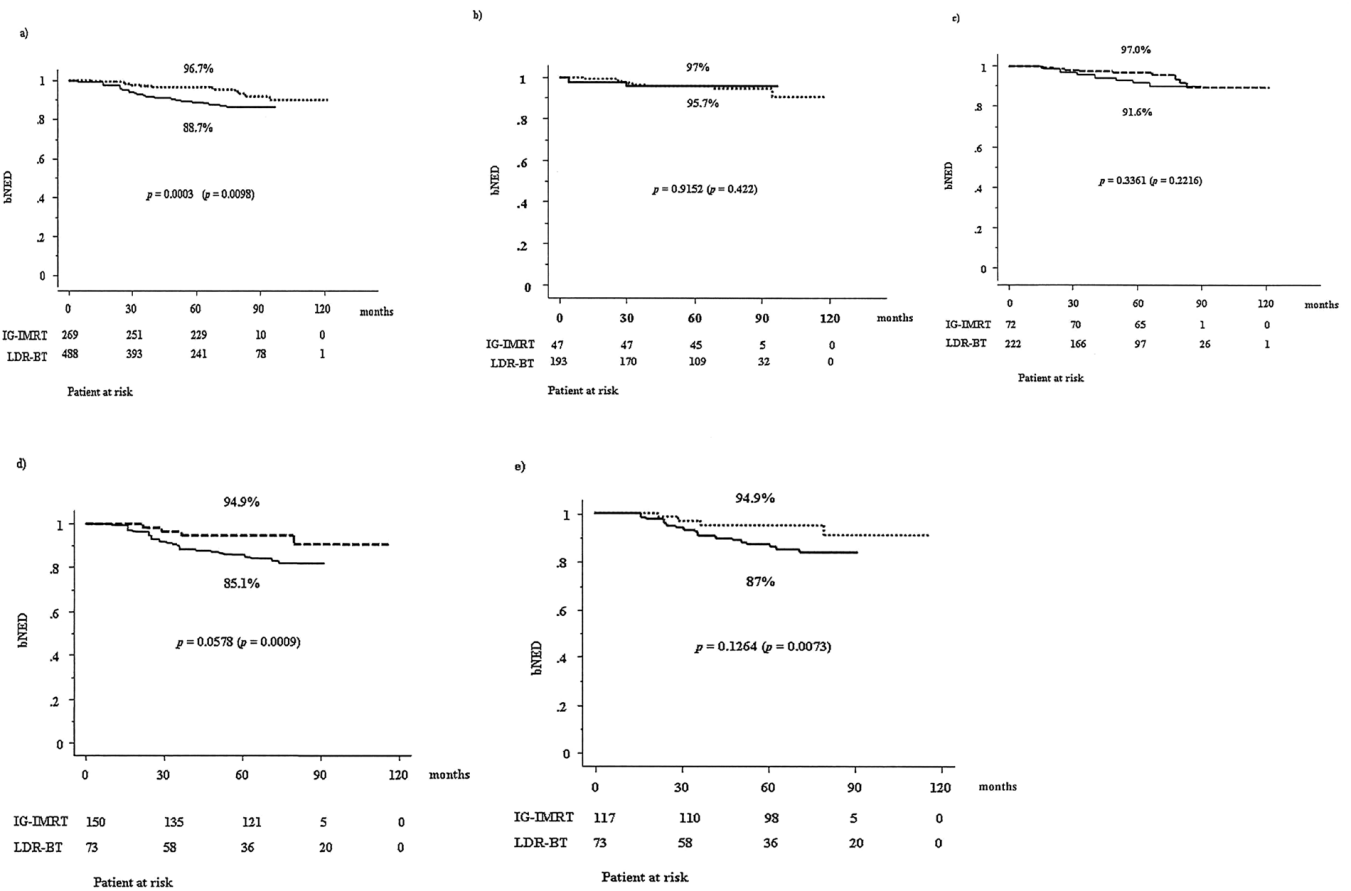
PSA control rates between LDR-BT with or without EBRT and IG-IMRT. (**a**) PSA control rates between LDR-BT and IG-IMRT in total population. (**b**) PSA control rates between LDR-BT and IG-IMRT in low risk group. (**c**) PSA control rates between LDR-BT and IG-IMRT in intermediate risk group. (**d**) PSA control rates between LDR-BT and IG-IMRT in high risk group. (**e**) PSA control rates between LDR-BT and IG-IMRT in selected high risk group excluding T3b-4 and/or iPSA > 50 ng/ml. Solid line depicted IG-IMRT and dotted line depicted LDR-BT with or without EBRT. bNED = no biochemical evidence of disease, p-values depicted in parenthesis’s were generated by Inverse probability of treatment weighting (IPTW) involving propensity scores was used to reduce background selection bias.

In the IG-IMRT group, 35 (13%) patients developed biochemical failure, compared with 20 (4.1%) in the LDR-BT group. The actuarial five-year biochemical failure-free survival rate was 88.7% (95% confidential interval, 85.4%–92.8%) and 96.7% (94.9%–98.5%, *p* = 0.0003, Fig. 1) in IG-IMRT and LDR-BT, respectively; it was 88.2% (85.1% for IG-IMRT and 94.9% for LDR-BT, *p* = 0.0578) for the high-risk group, 95.2% (91.6% and 97.0%, *p* = 0.3361) for the intermediate IG-IMRT, and 96.8% (95.7% and 97%, *p* = 0.8625) for the low-risk group. There is a significant difference in the biochemical control rate among those three risk groups (*p* = 0.0004). As shown in Table 2, the predictors of biochemical control on univariate analysis included treatment (LDR-BT vs. IG-IMRT), T classification (T1-2 vs. T3-4), Gleason score (−7 vs. 8−), and a higher baseline PSA (−20 vs. 20<). On multivariate Cox regression analysis, the use of LDR-BT and T classification remained significant for improving PSA control (Table 2). As we found a borderline significance in the high-risk group, we also compared PSA control rate in the high-risk group excluding T3b and T4 diseases or iPSA 50 ng/ml or more (Fig. 1d), which also showed superior (but not statistically significant) tendency in LDR-BT than IG-IMRT groups. IPTW involving propensity scores was used to reduce background selection bias. IPTW showed a statistically significant difference between LDR-BT and IG-IMRT in total population (p = 0.0098), high risk (p = 0.0009), and high risk excluding T3-4 or initial PSA > 50 ng/ml group (p = 0.0073)(Fig. 1).

**Table 2:** Univariate and multi-variate analysis for PSA cotrol rate using Cox proportional hazrds model.

Variable	Strata	PSA control					
		Univariate analysis			Multivariate analysis		
		HR	95% CI	*p*	HR	95% CI	*p*
Age, years	<72	1	(referent)	—			
	72−	1.238	0.726–2.114	0.4332	NA		
T classification	T1-2	1	(referent)	—	1	(referent)	—
	T3-4	3.529	1.970–6.322	**<0.0001**	2.182	1.022–4.658	**0.0437**
Gleason score	−7	1	(referent)	—	1	(referent)	—
	8−	2.149	1.223–3.778	**0.0078**	1.6	0.610–2.205	0.6503
Pretratment PSA (ng/mL)	<20	1	(referent)	—	1	(referent)	—
	20−	2.677	1.437–4.989	**0.0019**	1.22	0.563–2.644	0.6147
D’Amico's risk classification	Low-Intermediate	1	(referent)	—	NA		
	High	2.719	1.598–4.624	**0.0002**			
Hormonal therapy	No	1	(referent)	—			
	Yes	1.313	0.766–2.251	0.3223	NA		
Treatment modalities	LDR-BT	1	(referent)	—	1	(referent)	—
	IG-IMRT	2.713	1.551–4.743	**0.0005**	1.962	1.034–3.720	**0.0391**

Bold values indicate statistically significance.

Abbreviations; CI = confidence interval; HR = hazard ratio.

For detailed comparison beteen LDR-BT alone and LDR-BT plus EBRT group, the actuarial five-year biochemical failure-free survival rate was 96.8% and 96.0% (p = 0.6073), respectively; it was 91.8% for LDR-BT alone and 97.0% for LDR-BT plus EBRT group (*p* = 0.1772) for high risk group, 97.4% and 94.1% (*p* = 0.9530) for the intermediate group, and 97.0% (LDR-BT only) for the low-risk group.

As there is no prostate-cancer-related death in this cohort, the five-year cause-specific survival rates were 100% in all groups.

The overall five-year survival rate was 98.5% (95% CI, 97.0%–100%) and 98.4% (97.1%–99.7%, *p* = 0.0139) in IG-IMRT and LDR-BT, respectively; it was 99.0% (97.9% for IG-IMRT and 98.6% for LDR-BT, *p* = 0.0373) for the high-risk group, 98.3% (100% and 97.4%, *p* = 0.9765) for the intermediate IG-IMRT, and 99.5% (97.9% and 99.3%, *p* = 0.3919) for the low-risk group. There are no statistically significant differences among those three risk groups (*p* = 0.1166) in overall survival rate.

For LDR-BT alone and LDR-BT plus EBRT group, overall survival rate was 98.8% and 96.0% (p = 0.3418), respectively; it was 100% for LDR-BT alone and 97.6% for LDR-BT plus EBRT group (*p* = 0.423) for high risk group, 98.0% and 92.3% (*p* = 0.3571) for the intermediate group, and 99.3% (LDR-BT only) for the low-risk group.

### Toxicity

Table 3 shows the incidence of late gastrointestinal (GI) and genitourinary (GU) toxicities. Grade 1, 2, and 3 late GI toxicities occurred in 30 (11%), 11 (4%), five (2%) patients in IG-IMRT and in 37 (8%), 11 (2%), and zero (0%) in LDR-BT, respectively (*p* = 0.0058). Late GU toxicity grades 1, 2, and 3 occurred in 36 (13%), 11 (4%), and one (0.4%) patients in IG-IMRT and in 178 (36%), 18 (4%), and two (0.4%) patients in LDR-BT (*p* < 0.0001), respectively. IG-IMRT showed more GI toxicity (*p* = 0.0023) and less GU toxicity (*p < *0.0001) than LDR-BT. The addition of EBRT to LDR-BT increased GI toxicities (Table 4).

**Table 3:** Comparison of late toxicities between IG-IMRT and LDR-BT.

Toxicities	Strata	IG-IMRT n = 269		LDR-BT n = 488		*p*-value
		No.	(%)	No.	(%)	
Gastrointestinal	0	233	(83%)	440	(89%)	**0.0023**
	1	30	(11%)	37	(8%)	
	2	11	(4%)	11	(2%)	
	3	5	(2%)	0	(0%)	
Genitourinary	0	221	(82%)	290	(59%)	**<0.0001**
	1	36	(13%)	178	(36%)	
	2	11	(4%)	18	(4%)	
	3	1	(0.4%)	2	(0.4%)	

IG-IMRT; image guided intensity modulated radiotherapy.

LDR-BT; low-dose-rate brachytherapy, EBRT: external beam radiotherapy, BT; brachytherapy.

**Table 4:** Comparison of late toxicities between between LDR-BT only and LDR-BT with EBRT.

Toxicities	Strata	BT only n = 420		BT+ EBRT n = 68		*p*-value
		No.	(%)	No.	(%)	
Gastrointestinal	0	390	(93%)	50	(74%)	**<0.0001**
	1	24	(6%)	13	(19%)	
	2	6	(1%)	5	(7%)	
	3	0	(0%)	0	(0%)	
Genitourinary	0	248	(59%)	42	(62%)	0.053
	1	153	(36%)	25	(37%)	
	2	17	(4%)	1	(1%)	
	3	2	(0.5%)	0	(0%)	

LDR-BT; low-dose-rate brachytherapy, EBRT: external beam radiotherapy, BT; brachytherapy.

For grade 3 GI toxicities, five patients treated with IG-IMRT showed rectal bleeding 6–36 months later (median 12 months) required hospitalization and/or tansfusion and/or laser ablation therapy.

For grade 3 GU toxicity, two LDR-BT only patients experienced two events (one obstratction 6 months and one incontinence 2 years 7 months) and one massive hematuria in IG-IMRT arm at 16 months occured.

## Discussion

Conventional treatment options for clinically localized prostate cancer include radical prostatectomy, EBRT, brachytherapy (low-dose rate or high-dose rate), and active surveillance in some situations^3^. On the basis of published evidence, conventionally fractionated IMRT is considered the standard of care over conventional three-dimensional conformal radiotherapy, which is an established modality for reducing the incidence of GI-adverse events^4^. A pioneer group study reduced GI toxicity from 13% using 3D-CRT to 5% using IMRT^9,10^. Furthermore, IGRT enabled us to deliver precise radiation exposure of the prostate, reducing uncertain exposure obtained using non-IGRT methods. Then, IG-IMRT has a potential to perform a higher-dose irradiation of the target lesion without unnecessary irradiation of normal tissues, and its use has increased in recent years; Spratt *et al*. reported the feasibility of higher dose of radiotherapy of 86.4 Gy^11^.

The role of brachytherapy is recognized to increase irradiated dose without morbidity, which proves the hypothesis that improving PSA control of prostate cancer translates into improving disease-specific and overall survival^7^. This concept had led many investigators to identify several ways to intensify local therapy with the use of ultra-high-dose escalation using both LDR-BT only or a combination of EBRT and brachytherapy^8^. In low and intermediate risk group, LDR-BT alone showed superior outcome than EBRT^7^. The Memorial Sloan Kettering group showed a better PSA control rate with EBRT plus brachytherapy than the 86.4 Gy of EBRT alone in the intermediate-risk group^12^.

Several retrospective and three phase III trials were undertaken to compare external beam radiotherapy with or without a brachytherapy boost (dose escalation). All phase III trials have demonstrated improvement in the PSA control rate with the addition of brachytherapy that spans all risk groups^12–17^. Most recently reported ASCEDE-RT, high- and unfavorable intermediate-risk disease were randomized to receive either 78 Gy IMRT or 46 Gy followed by LDR-BT boost^14^. In the 276 high-risk patients, the absolute benefit of an LDR boost for bNED at nine years was 20% (78% vs. 58%, *p* = 0.05). We also observed 8% gains in the PSA control rate at five years by LDR-BT than IG-IMRT, which remained at 8% in selected high-risk group excluding T3b-4 diseases and iPSA 50 ng/ml or more but not in intermediate- and low-risk groups. The role of LDR-BT therefore would be enhanced in the selected high-risk group. In several literatures, dose escalation added improvement in all risk groups; however, it is not the case in our population, all of whom experienced a good outcome. Even for the high-risk group, bNED 85.1% (and no disease-specific death) of IG-IMRT is a good outcome in comparison to other modalities.

The rates of failing definitive therapy are markedly different across risk groups and range from less than 5% in low-risk patients to 15% of high-risk patients^8^. Biochemical recurrence has been viewed as a poor surrogate for overall survival for prostate cancer patients. For high-risk patients only, biochemical recurrence is closely linked to the need for salvage therapies that can greatly impact quality of life in the short term and slow the progress to lethal diseases in a significant proportion of failures. The impact of primary treatment on overall survival is often disguised by the use of salvage hormonal therapy as there can be a long duration of response, with a median time to castration-resistant disease of seven years after radiotherapy^8^. For the patient whose life expectancy is less than 10 years, invasive procedure with morbidity will have limited its value to select. From the current clinical outcome, low to intermediate-risk group could be treated by each of those treatments. In selected younger high-risk groups, however, controversy remains because no clear overall survival benefit has been approved, and the patients would benefit from fully informed the merit that does intensified treatment such as LDR-BT, could improve PSA control.

Almost all IG-IMRT series reported up to 5% gastrointestinal G2≤ toxicity^4,17,18^, which conform to our data (4.4%), and LDR-BT showed much less G2≤ toxicity (2%). For GU toxicity, LDR-BT showed more toxicities G1≤ but equivocal 4.4% G2≤ toxicity which concurred to IG-IMRT. In the LDR-BT subgroup, EBRT + BT also showed increased toxicity in GI, which confirmed the risk of EBRT increasing GI toxicity^19^.

There are several limitations to our analysis. First, as this analysis was retrospective and not a randomized control trial, further validation by means of external data is required. Second, we did not examine other factors (i.e., hormonal therapy, diabetes mellitus, other drug usage, and performance status) that may influence PSA control rate. At present, however, we reached no concrete conclusion for the use of hormonal therapy in high BED radiotherapy, because a randomized trial to confirm the role of hormonal therapy was performed with up to 70 Gy of EBRT^3,10^. Third, a patients’ quality of life assessment would be useful as would essential information to select modalities^10,20^. Further investigation is certainly warranted. Finally, although we confirmed the safety and usefulness of our IG-IMRT schedule^5,6^, it is at present not a standard schedule especially for intermediate- to high-risk patients^3,21^. We have therefore elevated the prescribed dose to 78 Gy/39 fractions after confirming the safety of our protocol^5,6^.

In conclusion, IG-IMRT and LDR-BT showed equivocal PSA control rates in the low- and intermediate-risk groups. For selected high-risk patients, LDR-BT showed more potential to improve PSA control rates than IG-IMRT.

## Methods

### Patients

We included 758 patients with stage T1–T4 N0M0 prostate cancer who were treated using IG-IMRT (n = 269) or LDR-BT (n = 488) from June 2005 to September 2013 in this study. All patients had histology-proven adenocarcinoma. Patients were staged according to the D’Amico’s risk classification. The median patient age was 71 (ranging from 51–86) years. Patients’ clinical characteristics are shown in Table 1. PSA failure was defined using the Phoenix definition (nadir, +2 ng/ml) or as the start of salvage hormonal therapy. Common Terminology Criteria for Adverse Events version 4.0 Toxicity was applied to toxicity analysis. All patients provided informed written consent. This study was conducted in accordance with the Declaration of Helsinki and ethics committee/Kyoto Prefectural University of Medicine institutional review board permission (permission code; ERB-C-926).

### Treatment planning

#### Image-guided intensity-modulated radiotherapy (IG-IMRT)

The detailed methods were described elsewhere^5,6^. In brief, approximately one week before treatment initiation, we obtained CT and magnetic resonance imaging (MRI) data for treatment planning. At this time, each patient followed instructions for rectal emptying and bladder filling to minimize the interfraction motion. Patients were placed in the supine position, and CT was performed with 2-mm slice thickness. MRI (T1w and T2w) and CT images were fused to aid meticulous radiotherapy planning. The clinical target volume (CTV) was defined for the prostate and proximal seminal vesicles and prostate only in the low-risk group (D’Amico’s: classification: stage, T1c; Gleason score, <7; and PSA, <10 ng/ml). In the initial 2.2 Gy/fraction schedule, the CTV–PTV expansion margin was 5 mm in all directions, not avoiding the rectum. Pelvic nodal irradiation was not used. Ninety-five percent of PTV (D95) received at least the prescribed dose of 74.8 Gy in 34 fractions (2.2 Gy/fraction, IG-IMRT), unless the tumor was low risk in which case a dose of 72.6 Gy in 33 fractions was used. After the schedule was modified, the prescribed dose was reduced to 74 Gy (D95) in 37 fractions for the high- and intermediate-risk groups and 72 Gy in 36 fractions for the low-risk group (2 Gy/fraction). The posterior CTV–PTV expansion margin was also reduced to 3 mm, and the rectal contour was omitted from PTV, except in cases where the tumor was located adjacent to the rectum. We employed a 2.2 Gy fraction schedule between June 2007 to June 2009 and a modified 2 Gy fraction schedule from June 2009 to September 2011^5,6^.

We defined the bladder and rectum as solid organs at risk. Rectal volumes were contoured on axial slices from the recto-sigmoid junction to the anal verge. Planning constraints were set for the rectum and bladder: 35% and 18% of the rectal volume received <40 Gy and <60 Gy, respectively, and 50% and 25% of the bladder volume received <40 Gy and <65 Gy, respectively.

#### Low-dose-rate brachytherapy (LDR-BT) with or without external beam radiotherapy (EBRT)

The implant technique was previously described in detail^22–24^. All patients underwent TRUS preplanning three to four weeks before implantation to determine the number of seeds. We performed intraoperative permanent I-125 implantation (The OncoSeed model 6711; General Electric Healthcare, Barrington, IL) using a modified peripheral loading method. We used combination therapy for T3 ≤ or Gleason score sum 8 ≤  or Gleason score sum 7 (4 + 3) cases (not for Gleason score sum 7 (3 + 4) cases). Our prescription dose for the CTV (prostate) was 145 Gy (LDR-BT alone) or 110 Gy (LDR-BT with EBRT). Detailed patients characteristics of each group were shown in Supplemental Table 1e. Inter-Plan version 3.4 (ELEKTA, Stockholm, Sweden) was used as the treatment planning system.

### Statistical analysis

StatView 5.0 statistical software and R stat package (for IPTW)^26^ were used for statistical analyses. Percentages were analyzed using the chi-square test, and Student’s t-test was used for normally distributed data. The Mann–Whitney U-test for skewed data was used to compare means or medians, and the Kaplan–Meier method was used to analyze PSA control. Cox’s proportional hazard model was used for uni- and multivariate analyses (variables with p < 0.1 in the univariate analysis were included in the multivariate analysis). *p* < 0.05 was considered as statistically significant.

Because the included patients were not randomized, unbalanced baseline characteristics could have led to selection bias and, hence, influence the decision to undergo LDR-BT or IG-IMRT. The propensity score is defined here as the probability of being assigned to LDR-BT or IG-IMRT given the patients characteristics. In the calculation of the propensity scores, the logistic regression model was used considering the baseline covariates shown in Table 2 (age, T classification, Gleason score, pretreatment PSA, and hormonal therapy). IPTW values were calculated from the propensity scores and represented the inverse probability of a participant receiving the observed treatment based on their characteristics. The treatment effects were recalculated using the IPTW with a Cox model. We weighted survival analysis using the inverse probability treatment weighting (IPTW) method, i. e., weighting patients who received LDR-BT by 1/propensity score, whereas patients who received IG-IMRT were weighted by 1/(1–propensity score).

## Electronic supplementary material

Supplementary information
