# Author Correction: Comparison of Image-Guided Intensity-Modulated Radiotherapy and Low-dose Rate Brachytherapy with or without External Beam Radiotherapy in Patients with Localized Prostate Cancer

**DOI:** 10.1038/s41598-018-32319-z

**Published:** 2018-09-20

**Authors:** Takuji Tsubokura, Hideya Yamazaki, Koji Masui, Naomi Sasaki, Daisuke Shimizu, Gen Suzuki, Satoaki Nakamura, Kei Yamada, Koji Okihara, Takumi Shiraishi, Ken Yoshida, Tatsuyuki Nishikawa, Haruumi Okabe

**Affiliations:** 1 Department of Radiology, Fukuchiyama City Hospital, 231 Atsunakamachi, Fukuchiyama, Kyoto Prefecture 620-8505 Japan; 2 Department of Radiology, Graduate School of Medical Science, Kyoto Prefectural University of Medicine, 465 Kajiicho Kawaramachi Hirokoji, Kamigyo-ku, Kyoto, 602-8566 Japan; 3 Urology, Graduate School of Medical Science, Kyoto Prefectural University of Medicine, 465 Kajiicho Kawaramachi Hirokoji, Kamigyo-ku, Kyoto, 602-8566 Japan; 4 Department of Radiology, Osaka Medical College, 2-7 Daigaku-machi, Takatsuki-City, Osaka, 569-8686 Japan; 5 Department of Radiology, Ujitakeda Hospital, Uji-city, Kyoto, Japan

Correction to: *Scientific Reports* 10.1038/s41598-018-28730-1, published online 12 July 2018

The version of this Article previously published contained lower resolution images for Figure 1. This has now been corrected in the PDF and HTML versions of the paper.

